# Resolvins as Regulators of the Immune System

**DOI:** 10.1100/tsw.2010.72

**Published:** 2010-05-04

**Authors:** Hiroyuki Seki, Takaharu Sasaki, Tomomi Ueda, Makoto Arita

**Affiliations:** ^1^ Department of Health Chemistry, Graduate School of Pharmaceutical Sciences, University of Tokyo, Japan; ^2^ Department of Anesthesiology, Keio University School of Medicine, Tokyo, Japan; ^3^ PRESTO Japan Science and Technology Agency, Saitama, Japan

**Keywords:** resolvin, lipid mediator, omega-3 fatty acid, EPA, DHA, inflammation, immune regulation, resolution of inflammation

## Abstract

Inflammation is the first response of the immune system to infection or injury, but excessive or inappropriate inflammatory responses contribute to a range of acute and chronic human diseases. Clinical assessment of dietary supplementation of ω-3 polyunsaturated fatty acids (i.e., eicosapentaenoic acid [EPA] and docosahexaenoic acid [DHA]) indicate that they have beneficial impact on these diseases, although the mechanisms are poorly understood at the molecular level. In this decade, it has been revealed that EPA and DHA are enzymatically converted to bioactive metabolites in the course of acute inflammation and resolution. These metabolites were shown to regulate immune cell functions and to display potent anti-inflammatory actions both *in vitro* and *in vivo*. Because of their ability to resolve an acute inflammatory response, they are referred to as proresolving mediators, or resolvins. In this review, we provide an overview of the formation and actions of these lipid mediators.

### INTRODUCTION

Epidemiological studies late in the 1970s revealed an association between diets containing fish oil rich in ω-3 polyunsaturated fatty acids (PUFAs) and reduced incidence of cardiovascular disease[1]. In the 1990s, the beneficial effect of supplementation of ω-3 PUFAs was firmly established by the GISSI-Prevenzione trial[2], in which the effect of ω-3 PUFAs was examined in 11,324 survivors of myocardial infarction. The trial showed that ω-3 PUFA supplementation reduced the incidence of sudden death by approximately 45%. Two mechanisms have been proposed to explain the actions of ω-3 PUFAs: (1) they prevent the conversion of arachidonic acid (AA) into proinflammatory eicosanoids, such as prostaglandins (PGs) and leukotrienes (LTs) via substrate competition, and (2) they serve as an alternative substrate to produce less potent 5-series LTs and 3-series PGs and thromboxanes (Fig. 1)[3,4,5,6,7]. Of interest, >80% of the individuals in the GISSI-Prevenzione trial were simultaneously receiving aspirin and ω-3 PUFAs. Aspirin blocks platelet activation by inhibiting cyclooxygenase-1 (COX-1)–dependent

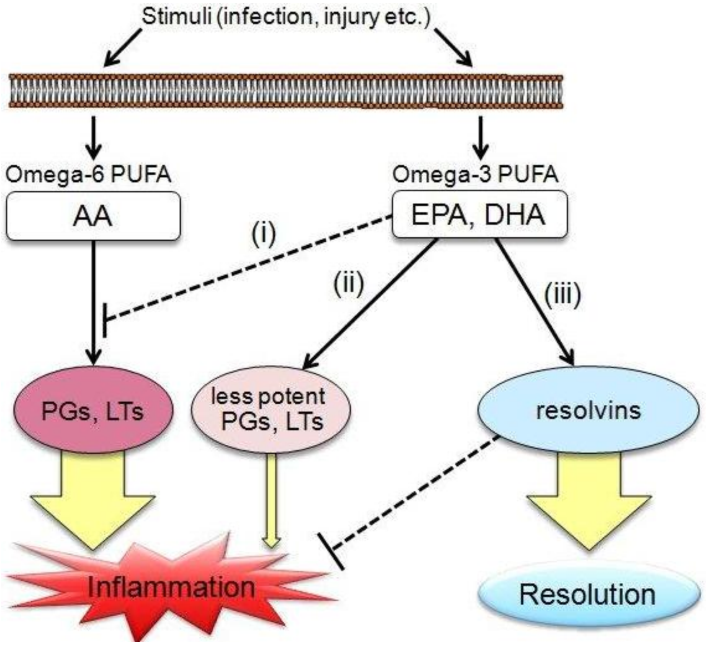

**FIGURE 1.** Possible mechanisms of the anti-inflammatory actions of ω-3 PUFAs. Omega-3 PUFAs are thought to act via several possible mechanisms: (1) preventing the conversion of AA into proinflammatory eicosanoids, such as 4-series LTs and 2-series PGs via substrate competition; (2) serving as an alternative substrate to produce less potent 5-series LTs and 3-series PGs and thromboxanes; and (3) converting EPA and DHA into bioactive metabolites, such as resolvins with anti-inflammatory and proresolving properties.

thromboxane formation, but also triggers the formation of epimeric forms of naturally occurring lipid mediators (i.e., 15-epi-lipoxin A4)[8]. The latter property of aspirin inspired the idea that aspirin promotes the formation of lipid mediators from ω-3 PUFAs. To examine this possibility, mice were given injections of sterile air to create air pouches, followed by intrapouch injections of tumor necrosis factor alpha (TNF-α) with ω-3 PUFAs and aspirin on board[9,10]. The inflammatory exudates formed in the air pouches contained several previously unrecognized products derived from eicosapentaenoic acid (EPA) and docosahexaenoic acid (DHA). These newly identified products were named resolvins because they were originally identified from mouse-resolving exudates[10]. Application of exogenous resolvins at nanomolar range inhibited polymorphonuclear leukocyte (PMN) migration across endothelial cell monolayers[10]. Also *in vivo*, intravenous administration of resolvins in nanogram quantities dramatically reduced PMN infiltration in a murine acute inflammation model[10]. Resolvins also regulate macrophage and dendritic cell functions by controlling the production of cytokines and/or by regulating phagocytosis[11,12].

### STRUCTURE AND FUNCTION OF RESOLVINS

Resolvins are a family of bioactive metabolites generated in response to inflammation by enzymatic conversion of EPA or DHA. The name resolvin (resolution phase interaction products) was introduced for these compounds because they were originally identified in the resolution phase of acute inflammation. They possess distinct structures consisting of di- or trihydroxyl groups with conjugated double bonds present in the molecule. EPA-derived resolvins are designated "E-series resolvins" and those from DHA are denoted "D-series resolvins".

### EPA-Derived Resolvins; E-Series Resolvins

Resolvin E1 (RvE1) was originally identified in the inflammatory exudates formed in murine air pouches via intrapouch injections of TNF-α with EPA and aspirin on board[9]. Instillation of TNF-α into the pouches that are created by injection of sterile air induces a PMN-rich inflammatory exudate that is maximal within 2–4 h and reaches PMN resolution by 24 h[13]. Administration of EPA and aspirin reduced PMN recruitment to the inflammatory site by 25–60%[9]. LC-MS/MS analysis of the exudate-derived materials revealed novel monohydroxy fatty acids, such as 18*R*-hydroxy-EPA (18*R*-HEPE), as well as a trihydroxylated compound (5,12,18-triHEPE). The biosynthetic pathway of this product was established *in vitro* (Fig. 2). Recombinant human COX-2 treated with aspirin generated 18*R*-HEPE, and its production was blocked by selective COX-2 inhibitors[9]. 18*R*-HEPE, when incubated with activated human PMNs, was converted to 5,12,18*R*-triHEPE via 5-lipoxygenase (LOX) activity. 5,12,18*R*-triHEPE had a potent anti-inflammatory activity in blocking PMN migration both *in vitro* and *in vivo*[9]. 5,12,18*R*-triHEPE was named RvE1 and the complete stereochemical assignment of RvE1 was established as 5*S*,12*R*,18*R*-trihydroxy-6*Z*,8*E*,10*E*,14*Z*,16*E*-EPE based on the matching of physical and biological properties with synthetic compounds[14].

In addition to RvE1, PMN exposed to 18-HEPE generated a dihydroxylated product (i.e., 5*S*,18-diHEPE) via 5-LOX, and was named RvE2 (Fig. 2)[15]. Intravenous administration of RvE2 reduced PMN infiltration in zymosan-induced peritonitis and its potency was almost equivalent to that of RvE1. Total organic synthesis and complete stereochemical assignment of RvE2 was established as 5*S*,18*R*-dihydroxy-6*E*,8*Z*,11*Z*,14*Z*,16*E*-EPE[16].

### DHA-Derived Resolvins; D-Series Resolvins

Lipidomic analysis of exudates in murine air pouches via intrapouch injections of TNF-α with DHA and aspirin revealed novel 17*R*-hydroxy-DHA (HDoHE) as well as 17*R*-containing di- and tri-HDoHEs[10]. The biosynthetic pathway was established *in vitro* (Fig. 3). Recombinant human COX-2 treated with aspirin converted DHA to 17*R*-HDoHE. 17*R*-HDoHE, when incubated with activated human PMNs, was converted to 17*R*-containing tri-HDoHE. These products were categorized as aspirin-triggered RvDs (AT-RvDs) to distinguish from 17*S*-RvDs that are synthesized in the absence of aspirin[10]. In the absence of aspirin, DHA was converted to 17*S*-HDoHE by 12/15-LOX activity, and then further converted to 17*S*-containing RvDs (Fig. 3)[10,17]. The complete stereochemistry of RvD1 was established as 7*S*,8*R*,17*S*-trihydroxy-4*Z*,9*E*,11*E*,13*Z*,15*E*,19*Z*-DHA, and RvD2 as 7*S*,16*R*,17*S*-trihydroxy-4*Z*,8*E*,10*Z*,12*E*,14*E*,19*Z*-DHA[18,19]. They both blocked transendothelial migration of human PMNs ($EC_{50}$ ~30 nM), and intravenously administered RvD1 or AT-RvD1 at the nanogram level strongly limited PMN infiltration in the murine zymosan-induced peritonitis model[18].

## RESOLVINS AS REGULATORS OF THE IMMUNE SYSTEM

Resolvins display protective actions against leukocyte-mediated tissue injury and regulate excessive proinflammatory responses *in vivo*. In this section, we give an overview of the bioactions of resolvins on immune systems both *in vitro* and *in vivo* (see also Tables 1 and 2).

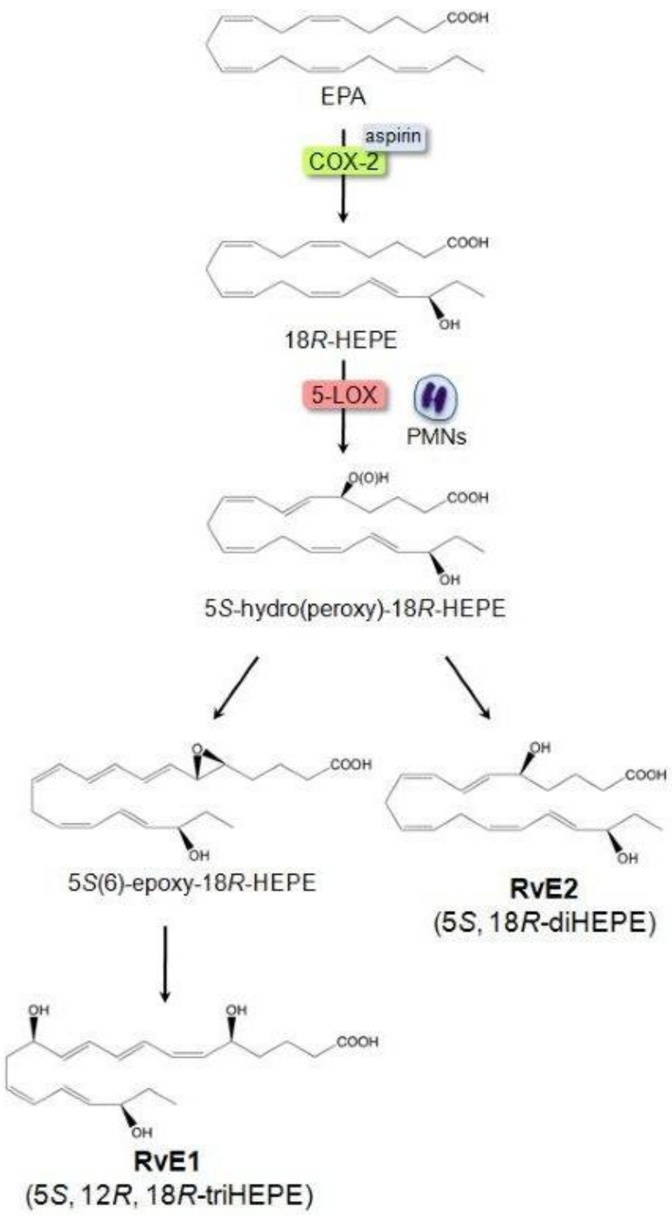

**FIGURE 2**. Biosynthetic scheme proposed for E-series resolvins. EPA is oxygenated at the carbon-18 position via the action of aspirin-acetylated COX-2 to form 18*R*-HEPE, which is subsequently oxygenated to form 5*S*-hydro(peroxy)-18*R*-HEPE via 5-LOX activity. The 5*S*-hydroperoxy group is either converted to an 5,6-epoxide intermediate to form RvE1 or reduced to a 5*S*-hydroxyl group to form RvE2.

### Target Cells of Resolvins

#### *Neutrophils (PMNs)*

PMNs constitute the first line of immune defense by migrating to sites of injury or infection. Transmigrating PMNs undergo a potent respiratory burst and degranulation in response to invading pathogens. When this reaction becomes excessive or inappropriate, it can cause tissue damage and contribute to numerous inflammatory diseases[20].

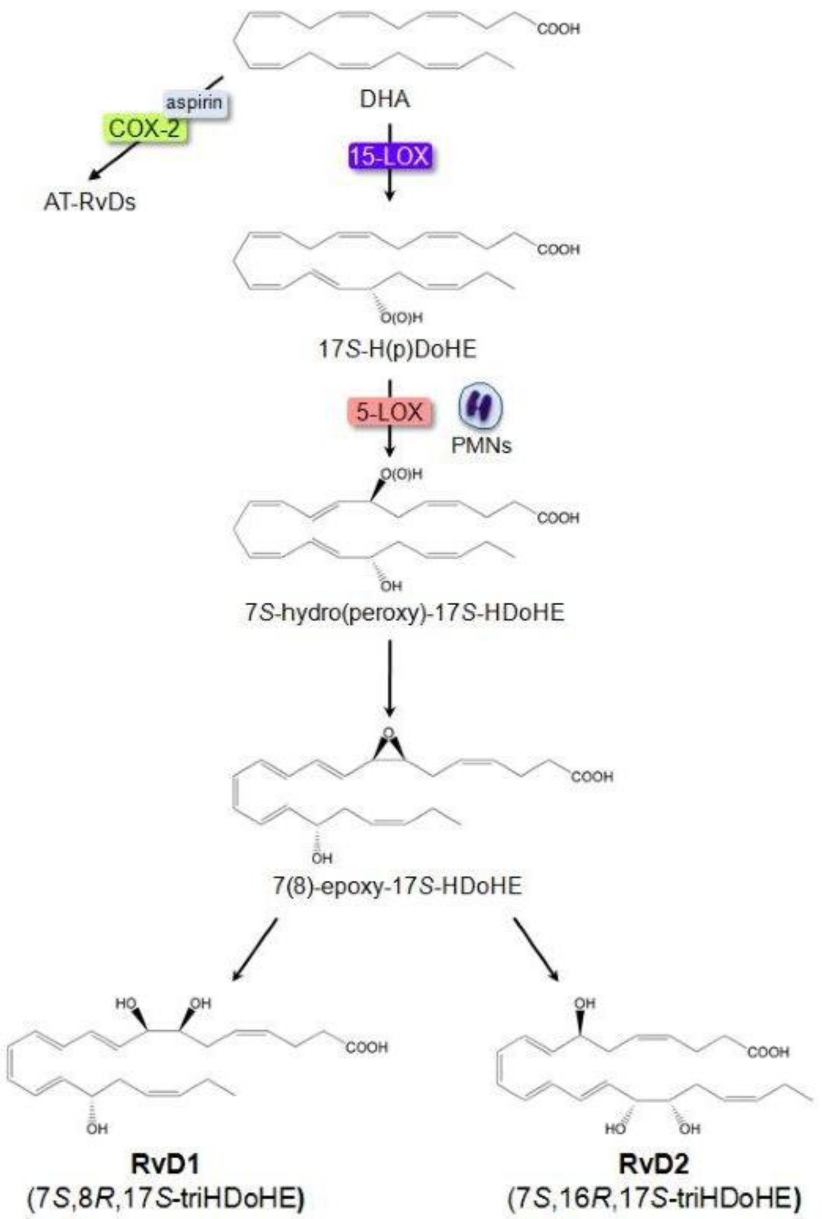

**FIGURE 3**. Biosynthetic scheme proposed for D-series resolvins. DHA is the endogenous substrate that is converted into RvDs. DHA is oxygenated at the carbon-17 position via actions of 15-LOX to form 17*S*-H(p)DoHE. This metabolite is further converted via sequential actions of leukocyte 5-LOX and leads to the formation of RvD1 and RvD2. DHA is also converted to 17*R*-HDoHE by aspirin-acetylated COX-2, which is further converted to aspirin-triggered (AT)-RvDs via 5-LOX activity.

Transendothelial migration is a pivotal event in PMN recruitment. RvE1 inhibited $LTB_4$-stimulated PMN migration across microvascular endothelial cell monolayers with an $IC_{50}$ of ~10 nM[9]. Also RvE1 inhibited $LTB_4$-stimulated PMN transmigration across a monolayer barrier consisting of choroid-retinal endothelial cells (CRECs)[21]. Transepithelial migration of PMNs represents an important component of the innate immune response and is a pathological hallmark of active mucosal inflammation. RvE1 potently attenuated f-MET-Leu-Phe (fMLP)–induced PMN transmigration across KB oral epithelial cells in a concentration-dependent manner[22]. RvE1 interacted with the $LTB_4$ receptor BLT1 that is expressed on PMNs with a Kd value of ~50 nM, and attenuated proinflammatory signals by $LTB_4$[23]. Resolvins also

**Table 1 E-series resolvins in cellular actions and disease models**

| Action | Reference(s) |
|---|---|
| **Target cells** | |
| **Neutrophil** | |
| · Stop $LTB_4$-stimulated transendothelial migration | 9, 21 |
| · Block fMLP-stimulated transepithelial migration | 22 |
| · Block IL-8-stimulated chemotaxis | 11 |
| · Enhance phagocytosis, ROS generation and pathogen killing | 11 |
| · Regulate L-selectin shedding and reduce CD18 expression | 29 |
| · Inhibit superoxide generation | 36 |
| **Dendritic cell** | |
| · Inhibit IL-12 production | 14 |
| · Inhibit the release of IL-23, IL-6 and TNF | 40 |
| · Decrease chemotaxis in response to CCL21 and CCL19 | 25 |
| **Macrophage** | |
| · Promote phagocytosis of apoptotic PMNs and zymosan | 12 |
| **Platelet** | |
| · Block ADP- and U46619- stimulated aggregation | 29 |
| **Endothelial cells** | |
| · Inhibit pro-inflammatory cytokine production and adhesion molecule mRNA expression | 35 |
| **Disease models** | |
| **Ocular angiogenesis** | |
| · Reduce pathologic neovascularization | 34, 35 |
| · Reduce PMN migration | 35 |
| · Reduce cytokine and VEGFs mRNA expression | 35 |
| **Periodontitis** | 36, 37 |
| · Reduce PMN infiltration | |
| · Prevent connective tissue and bone loss | |
| · Promote healing of diseased tissues | |
| · Regenerate lost soft tissues and bone | |
| **Asthma** | 40 |
| · Dampen airway inflammation and hyperresponsiveness | |
| · Regulate cytokine and lipid mediator production | |
| **Aspiration pneumonia** | 49 |
| · Increase survival | |
| · Increase bacterial clearance | |
| · Inhibit PMN infiltration | |
| · Decrease NF-κB (p65) activation and pro-inflammatory mediator production | |
| **Obesity** | 52 |
| · Alleviate obesity-induced insulin resistance and advanced hepatic steatosis | |
| **Peritonitis** | |
| · Reduce PMN infiltration | 12, 15, 41 |
| · Increase mononuclear cell | 12 |
| · Enhance lymphatic removal of phagocytes | 12 |
| **Inflammatory bowel disease** | 58 |
| · Increase survival and improve histological damage | |
| · Reduce PMN infiltration and pro-inflammatory gene expression | |

**Table 2 D-series resolvins in cellular actions and disease models**

| Action | Reference(s) |
|---|---|
| **Target cells** | |
| **Neutrophil** | |
| · Stop $LTB_4$- and fMLP-stimulated transendothelial migration | 35 |
| · Block IL-8 and PAF-stimulated chemotaxis | 19 |
| · Inhibit superoxide and ROS generation | 19 |
| **Macrophage** | 19 |
| · Promote phagocytosis of apoptotic cells, zymosan and bacteria | |
| **Endothelial cells** | |
| · Inhibit pro-inflammatory cytokine production and adhesion molecule mRNA expression | 35 |
| · Stimulate the production of prostacyclin and nitric oxide | 19 |
| **Disease models** | |
| **Ocular angiogenesis** | |
| · Reduce pathologic neovascularization | 34, 35 |
| · Reduce PMN migration | 35 |
| · Reduce cytokine and VEGFs mRNA expression | 35 |
| **Ischemic acute kidney injury** | 55 |
| · Reduce PMN and monocytes infiltration | |
| · Decrease postischemic increase in serum creatinine and kidney fibrosis | |
| **Peritonitis** | 18, 19 |
| · Reduce PMN infiltration | |
| **Microbial sepsis** | 19 |
| · Increase survival | |
| · Increase macrophage phagocytosis | |
| · Decrease local and systemic bacterial burden | |
| · Decrease PMN infiltration and pro-inflammatory cytokine production | |

enhanced the effecter functions of PMNs. RvE1 (10 nM) enhanced PMN phagocytosis and killing of *Candida albicans*[11]. RvD2 (10 nM) also enhanced PMN phagocytosis of *Escherichia coli* that was accompanied by an increase in intracellular reactive oxygen species generation[19]. These actions of resolvins are important for limiting invasion of pathogens and resolving inflammation.

### Dendritic Cells (DCs)

DCs are immune cells that have essential roles in innate immunity and in the initiation of adaptive immunity[24]. In their immature state, DCs are distributed in tissues that are in contact with the external environment, such as the mucosal surfaces or the skin. During inflammation, they migrate into inflamed peripheral tissues where they capture antigens. After they mature, they migrate to lymph nodes where they activate naive T cells and provide cytokines required for T-cell proliferation/differentiation. Intraperitoneally administered Soluble *Toxoplasma gondii* tachyzoite antigen (STAg) activated the mobilization of splenic DCs to T-cell enriched areas and production of IL-12. Treatment with RvE1 blocked DC migration and IL-12 production triggered by STAg[14].

A major mechanism for resolving an inflammatory process is the deletion of effector T cells. RvE1 enhances the resolution by inducing the apoptosis of T cells. Bone marrow–derived DCs exposed to RvE1, especially during differentiation, maintained an immature chemokine receptor pattern following lipopolysaccharide (LPS) stimulation, i.e., continued to express CCR5 and did not up-regulate CCR7.

This response was consistent with the migration pattern, i.e., DCs exposed to RvE1 did not migrate in response to CCL19/21 and strongly migrated toward CCL3/5[25]. In addition, RvE1 induced apoptosis of antigen-specific activated $CD4^+$ T cells through inducing DC expression of indoleamine 2,3-dioxygenase[25], which catabolizes the amino acid tryptophan and plays an important role in T-cell apoptosis[26]. These results imply that DCs exposed to RvE1 and pathogens remain at the inflammatory site instead of migrating to lymph nodes, and induce apoptosis of effecter T cells at the inflammatory site.

### Macrophages

The key histological event in tissue resolution is the clearance of apoptotic PMNs and tissue debris[27]. *In vitro*, RvE1 (100 nM) stimulated murine peritoneal macrophages to ingest apoptotic PMNs[12]. RvE1 interacts with ChemR23, which is expressed in macrophages as well as DCs[14]. RvE1 induced human monocyte–derived macrophages to ingest opsonized zymosan, and this enhancement was blocked by an antibody against ChemR23[28]. RvE1 stimulated Akt and ribosomal S6 phosphorylation signals in macrophages[28]. *In vivo*, in zymosan-induced peritonitis, RvE1 (300 ng/mouse) given at the peak of inflammation enhanced the removal of zymosan by phagocytes and its transport to lymph nodes and spleen[12]. These results indicated that RvE1 helped to resolve inflammation by activating macrophages to clear apoptotic cells and debris from inflammatory sites.

In mice with microbial sepsis initiated by cecal ligation and puncture, RvD2 treatment (100 ng/mouse) decreased the bacterial burden in both the blood and peritoneum, and promoted the clearance of bacteria[19]. Evidence for direct macrophage actions was obtained *in vitro*, where RvD2 (1 nM) enhanced macrophage phagocytosis of opsonized zymosan[19]. These results indicated that RvD2 stimulated the resolution of inflammation by activating macrophages to clear bacteria.

### Platelets

Platelets play important roles in blood coagulation, wound healing, and inflammation. Platelet aggregation, which has further effects on platelet-leukocyte and platelet-endothelium interactions, is essential in thrombosis. These active cell-cell interactions are responsible for and provide the connection between thrombosis and inflammation. RvE1 at nanomolar range blocked ADP-stimulated platelet aggregation in a concentration-dependent manner[29]. In addition to ADP, RvE1 also blocked platelet aggregation stimulated by U46619 (thromboxane receptor agonist), with similar kinetics[29]. Of interest, RvE1 did not block collagen-induced platelet aggregation. These results suggest that RvE1 acts to block excessive platelet aggregation rather than disrupt physiologic coagulation induced by collagen.

### Leukocyte-Endothelial Cell Interactions

Leukocyte recruitment is a key step in acute inflammation. The interaction between leukocytes and the endothelium comprises a variety of adhesive and migratory molecular events including low-affinity transient and reversible rolling adhesions, integrin-dependent firm adhesive interactions, and migration of leukocytes thorough the endothelium[30,31]. Intravital microscopy demonstrated that intravenous administration of RvE1 (100 ng) rapidly reduced leukocyte rolling by approximately 40% in cremaster muscle venules of mice[29]. RvD2 (1 nM) decreased platelet activating factor (PAF)–stimulated leukocyte adherence and emigration in the microcirculation in the cremaster muscle of mice. RvE1 inhibited $LTB_4$-stimulated PMN migration across microvascular endothelial monolayers with an apparent $IC_{50}$ of ~10 nM[9]. RvD2 reduced PAF and complement-stimulated capture and adhesion of PMNs by human umbilical vascular endothelial cells (HUVECs) under flow[19]. These actions of resolvins are mediated in part by modulating the expression of adhesion molecules on leukocytes and the production of antiadhesive mediators by endothelial cells. Addition of RvE1 (30 nM) to whole blood reduced CD18 expression on both PMNs and monocytes approximately by approximately 50%[29]. RvD2 diminished PAF-stimulated CD62L (L-selectin) shedding on isolated human PMNs and CD18 surface expression[19]. Topical administration of RvD2 (100 pg/ear) increased endothelial-dependent nitric oxide production[19]. Corroboratory results were obtained with HUVECs, whereby RvD2 dose dependently stimulated nitric oxide generation[19].

### Impact of Resolvins in Disease Models

#### *Ocular Angiogenesis Models*

While neovascularization is an essential biological process, there are many instances where the growth of new vessels can have pathogenic consequences. Neovascularization in the oculus is a major component of several ocular disorders, including diabetic retinopathy, retinopathy of prematurity, age-related macular degeneration, and corneal disease[32]. A popular model for studying abnormal angiogenesis in the retina is the oxygen-induced retinopathy model in mice[33]. Exposure of the neonatal mouse eye to hyperoxia leads to obliteration of capillaries and upon return to room air, results in retinal ischemia and proliferative vascular disease in the retinal vasculature[33]. Intraperitoneal administration of RvD1 or RvE1 (10 ng/day) conferred significant protection from vaso-obliteration and neovascularization compared with the control[34].

Placement of intracorneal sutures in the mouse cornea causes inflammation of the cornea and leads to corneal neovascularization. Subconjunctivally injected RvE1 or RvD1 at 100 ng inhibited PMN migration into the inflamed cornea and reduced mRNA levels of IL-1β and TNF-α, which are known to enhance the expression of angiogenic factors[35]. RvE1 and RvD1 also reduced the mRNA expressions of VEGF-A, VEGF-C, and VEGFR2, which play a pivotal role in vascular development in causing inflamed corneas, and as a result, significantly reduced neovascularization in the inflamed cornea[35].

#### *Periodontal Disease Model*

Periodontal disease is a local inflammatory disease characterized by PMN-mediated tissue injury followed by development of a chronic lesion. Periodontitis was induced in rabbits by topical application of *Porphyromonas gingivalis* to the ligated teeth every other day for a 6-week period. Topical application of RvE1 (4 μg/tooth, three times per week) at the ligature inhibited >95% of alveolar bone loss[36]. Histological analysis demonstrated prominent leukocyte infiltration and bone loss in vehicle-treated animals, whereas much less PMN infiltration or tissue damage was observed in RvE1-treated animals. In addition, staining the sections with tartrate-resistant acid phosphate (TRAP), an osteoclast marker, showed that tissues from RvE1-treated animals had fewer TRAP-positive cells than tissues from vehicle-treated animals. In addition, RvE1 restored the lost bone and ameliorated clinical periodontal disease parameters, including pocket depth, infrabony defect depth, and tooth mobility[37].

#### *Asthma Model*

Asthma is a chronic inflammatory disease of the airways characterized by infiltration of eosinophils and T lymphocytes that produce cytokines and lipid mediators that contribute to disease pathogenesis[38,39]. Allergic airway inflammation was induced in mice by sensitization with intraperitoneal injection of ovalbumin (OVA), followed by repeated aerosol challenge with OVA[40]. In this model, airway leukocyte trafficking was spontaneously resolved within 1 week after induction of airway inflammation. Intravenous administrations of RvE1 (100 ng/mouse for 3 days) shortened the resolution interval (time required for inflammatory cell numbers to reach 50% of the numbers noted at the peak inflammation[41]), and ameliorated airway mucus and hyper-responsiveness, two typical symptoms of asthma. Treatment with RvE1 decreased production of proinflammatory cytokines, such as IL-23, IL-6, and IL-17, and increased production of the counter-regulatory mediator interferon gamma (IFN-γ) in bronchoalveolar lavage fluid. In contrast, RvE1 had no apparent effect on IL-10 or the T-helper type 2 ($T_H2$) cytokines IL-4, IL-5, and IL-13. These cytokine alterations were reflected in shifting percentages of $T_H$-17 and IFN-γ–producing $CD4^+$ T cells in lung, lung-draining mediastinal lymph nodes, and spleen. During the resolution of allergic airway inflammation, exogenous RvE1 resulted in increased production of $LXA_4$ and reduced production of $LTB_4$.

### Aspiration Pneumonia Model

Aspiration of gastric contents into the airway causes intense parenchymal inflammation and PMN-dependent lung injury[42,43,44], and can predispose the host to bacterial pneumonia due to transient disruption in mucosal host defense mechanisms[45,46,47,48]. A murine aspiration pneumonia model was established by sequential intratracheal administration of hydrochloric acid and *E. coli*[49]. Intravenous administration of RvE1 (100 ng) prior to acid injury dramatically decreased lung PMN accumulation by 55% and enhanced clearance of *E. coli* from the lung[49]. RvE1 decreased aspiration pneumonia–induced nuclear factor kappa beta (NF-κB) (p65) activation by 40% and up-regulation of cytokines and chemokines, including IL-1β, IL-6, HMGB-1, MIP-1α, MIP-1β, KC, and MCP-1 in the lung. In addition, treatment with 100 ng RvE1 markedly improved survival in an aspiration pneumonia model even when it was given after the onset of pneumonia[49].

### Obesity Model

Obesity is characterized by the activation of an inflammatory process in metabolically active sites such as adipose tissue, liver, and immune cells[50]. The consequence of these responses are sharp increases in circulating levels of proinflammatory markers and modified insulin signaling, resulting in the development of insulin resistance. Mice with inactivating mutations in the leptin gene are hyperphagic, obese, hyperinsulinemic, and hyperglycemic, and have been used as a model for diabetes and obesity[51]. An ω-3 PUFA–enriched diet alleviated obesity-induced insulin resistance and advanced hepatic steatosis in *ob/ob* mice[52]. These beneficial effects were associated with up-regulation of genes involved in insulin sensitivity (i.e., PPARγ), glucose transport (i.e., GLUT-2 and GLUT-4), insulin receptor signaling (i.e., IRS-1 and IRS-2), and induction of adiponectin, an anti-inflammatory, antisteatoic, and insulin-sensitizing adipokine that is present in adipose tissue. Intraperitoneal administration of RvE1 (1.2 ng/g body weight every 24 h for 4 days) also elicited significant insulin-sensitizing effects by inducing adiponectin, GLUT-4, IRS-1, and PPARγ expression in adipose tissue[52].

### Peritonitis Model

Intraperitoneal administration of zymosan A, a cell wall component of yeast, leads to peritoneal leukocyte activation and inflammatory responses[53,54]. Intravenous administration of 100 ng RvE1/mouse reduced PMN infiltration into the peritoneum by approximately 45%. RvE1 and RvE2 were equally effective in reducing PMN infiltration[15]. Intravenous administration of RvD1 and AT-RvD1 both dose dependently reduced PMN infiltration with similar potency and efficacy (~35% reduction at 100 ng/mouse)[18]. RvD2 had a potent effect on peritonitis, causing a drastic (~70%) reduction in PMN infiltration[19]. In addition, RvE1 given at initiation or at peak of inflammation (300 ng/mouse i.p.) promoted resolution of acute peritonitis by enhancing clearance of leukocytes carrying zymosan into the lymphatics[12].

#### Ischemic Acute Kidney Injury Model

Ischemic injury to the kidney is characterized by renal tubular epithelial cell injury and inflammation. Renal ischemia followed by reperfusion (I/R) triggered the endogenous formation of DHA-derived RvD1 and 17*S*-HDoHE in the kidney tissue[55]. Exogenous administration of DHA to mice exposed to kidney I/R increased the amounts of these mediators in both plasma and renal tissue. Mice treated with 17*S*-HDoHE or RvD1 had lowered PMN infiltration in the kidney and lower plasma creatinine levels after ischemic injury than mice treated with vehicle alone. RvDs limited the deposition of interstitial collagen and therefore protected against fibrosis[55]. It should be noted that administration of RvDs after reperfusion still provided functional protection of the kidney as measured by serum creatinine levels.

#### Inflammatory Bowel Disease Model

Inflammatory bowel diseases, such as Crohn's disease and ulcerative colitis, are chronic and relapsing inflammatory disorders. They are characterized by abnormalities in mucosal responses to normally harmless bacterial antigens, resulting in abnormal cytokine production and persistent inflammation leading to mucosal damage[56,57]. Pathologically, inflammatory bowel disease is characterized by colon inflammation associated with loss of barrier function, leukocytosis, and proinflammatory gene expressions. RvE1 protected against severe inflammatory bowel disease induced by 2,4,6-trinitrobenzenesulfonic acid (TNBS)[58]. Intraperitoneal administration of RvE1 (1 μg/mouse) 8, 1, and 0 days before induction of colitis increased survival rates, sustained body weights, and improved histological scores. TNBS-induced colitis is characterized by massive infiltration of PMNs and macrophages, producing high levels of proinflammatory cytokines in the early stages, followed by $CD4^+$ T-cell infiltration. Treatment with RvE1 decreased PMN numbers in the mucosal tissues, reduced the expressions of proinflammatory genes, including TNF-α, IL-12, inducible nitric oxide synthase, and COX-2 in the inflamed colons, and also reduced serum anti-TNBS IgG levels.

#### Sepsis Model

Sepsis is defined as a systemic inflammatory response syndrome in the presence of an infection and is a major cause of death in intensive care units[59]. The prevalent hypothesis is that sepsis and septic shock are caused by an excessive defense and inflammatory response characterized by massive increases in reactive oxygen species, nitric oxide, and inflammatory cytokines. In mice with microbial sepsis initiated by cecal ligation and puncture (CLP), intravenous administration of 100 ng RvD2 sharply decreased the amount of live bacteria in both the blood and peritoneum[19]. This was associated with a significant reduction in total leukocytes migrating into the peritoneum. Treating CLP-operated mice with RvD2 (1) dramatically increased survival rates; (2) protected against hypothermia; (3) restored activity levels at 12 h post-CLP; (4) promoted phagocyte-dependent bacterial clearance in lymph nodes; (5) drastically reduced plasma levels of proinflammatory cytokines, such as IL-6, IL-1β, IL-23 and TNF-α, which are associated with poor outcomes in sepsis; and (6) decreased proinflammatory mediators, including $PGE_2$ and $LTB_4$, in the peritoneum. These multilevel anti-inflammatory and proresolving actions of RvD2 explain the increased survival from sepsis induced by CLP.

### CONCLUSIONS

Omega-3 PUFA–derived lipid mediator resolvins regulate immune systems by controlling cellular functions. EPA-derived E-series resolvins (i.e., RvE1 and RvE2) and DHA-derived D-series resolvins (RvD1 and RvD2) have potent anti-inflammatory and proresolution properties. They block excessive inflammatory responses and promote resolution by enhancing clearance of apoptotic cells and debris from inflamed tissues. These actions may underlie the beneficial impact of ω-3 PUFAs in human health and disease. Assessing spatial and temporal distributions of these bioactive lipid mediators by quantitative lipidomic analysis and elucidation of molecular mechanisms of action will advance our understanding of the role of resolvins as principal regulators of immune system in inflammation and host defense.

### ACKNOWLEDGMENT

M.A. is supported by the Japanese Science and Technology Agency PRESTO.
